# Poly ADP‐ribosylation regulates Arc expression and promotes adaptive stress-coping

**DOI:** 10.1007/s00213-025-06744-8

**Published:** 2025-01-14

**Authors:** Eliyahu Dahan, Leah Pergamenshik, Tze’ela Taub, Arthur Vovk, Jade Manier, Raphael Avneri, Elad Lax

**Affiliations:** Department of Molecular Biology, Ariel University, Ariel, Israel

**Keywords:** Poly ADP-ribosylation, Acute stress, Immediate-early genes, Stress adaptation, Activity-dependent genes, Stress coping

## Abstract

**Rationale:**

Rapid adaptation to stressful events is essential for survival and requires acute stress response and stress-coping strategy. However, the molecular mechanisms that govern this coping strategy have yet to be fully discovered.

**Objectives:**

This study aims to investigate the effects of poly ADP-ribosylation (PARylation) on stress-coping strategies following acute stress and to identify the target genes influenced by Parp1-induced histone PARylation.

**Methods:**

Mice were subjected to a forced swim test, a well-established acute stress paradigm, to evaluate cortical PARylation and assess the expression of activity-dependent genes. The pharmacological inhibition of Parp1 was conducted using ABT888 (Veliparib) to determine its effects on stress-coping behavior and related molecular changes.

**Results:**

The forced swim test increased cortical PARylation and upregulated the expression of activity-dependent genes. Systemic inhibition of Parp1 with ABT888 led to impaired stress-coping behavior, evidenced by a reduced immobility response during a subsequent forced swim test done 24 hours later. This impairment was associated with decreased chromatin PARylation and histone H4 acetylation at the Arc promoter and reduced Arc expression observed one hour after Parp1 inhibition.

**Conclusion:**

Our findings indicate that chromatin PARylation at the Arc promoters regulates histone H4 acetylation and Arc gene expression, and a subsequent impact on successful stress-coping behavior in response to acute stress.

**Supplementary Information:**

The online version contains supplementary material available at 10.1007/s00213-025-06744-8.

## Introduction

Long-term memory formation of stressful events is an adaptive mechanism enabling the optimization of coping strategies in the future. However, under abnormal circumstances, highly stressful events and persistent reactivation of said memory can lead to undesired outcomes such as avoidance behavior and post-traumatic stress disorder (Ressler et al. 2022). The formation of long-term memories requires *de-novo* protein synthesis preceded by time-dependent transcriptional alteration (Alberini 2008; Bisaz et al. 2014; Dudai & Eisenberg 2004; Eric R. Kandel 2001).

Poly ADP-ribosylation (PARylation) is a post-translational protein modification mostly known for its role in DNA repair. PARylation also promotes gene expression, presumably through increasing chromatin accessibility by evicting the linker histone H1 (Azad et al. 2018; Fontán-Lozano et al. 2010). The enzyme Poly (ADP-ribose) polymerase 1 (Parp1) is the most abundant member of the PARP family in mammals (Homburg et al. 2000; Ray Chaudhuri & Nussenzweig 2017). Parp1 catalyzes the polymerization of ADP-ribose, sourced in nicotinamide adenine dinucleotide (NAD +), to yield linear or branched structured poly ADP-ribose polymers (PAR).

Previous findings by us and others indicate that Parp1 is necessary for forming and retrieving long-term memories of feeding, stress, and cocaine-seeking behaviors (Cohen-Armon et al. 2004; Goldberg et al. 2009; Scobie et al. 2014; Visochek et al. 2016; Lax et al. 2017). PARylation is a rapid and transient posttranslational modification that occurs within 10–15 min after neuronal activity, promotes immediate-early gene expression and synaptic plasticity (Cohen-Armon et al. 2004; Homburg et al. 2000; Visochek et al. 2005, 2016). PARylation of Parp1 in the cortex and hippocampus is induced by a variety of signals via signal transduction mechanisms causing Parp1 activation by intra-molecular modifications exposing the NAD binding site in Parp1 (Cohen-Armon 2022). The PARylation of histone H1 regulates chromatin de-condensation and enables memory consolidation. However, it is still not clear which downstream genes are upregulated following histone PARylation. Stressful events that cause neuronal activity trigger the expression of immediate-early and activity-dependent genes required for further neuronal activation, memory formation, and neuroplasticity (Flavell & Greenberg 2008; Guzowski et al. 2001; Hernández et al. 2009; Bisaz et al. 2014; McEwen et al. 2015). We hypothesize that a forced swim test (FST), a highly stressful event, will initiate a fast histone PARylation alongside the mRNA expression of immediate-early and activity-dependent genes. We further speculated that systemic pharmacological inhibition of Parp1 before the FST will impair memory formation. This Parp inhibition will also reduce the mRNA expression of some known neuronal activity-related, immediate-early, and activity-dependent genes, thus allowing us to identify the mRNA expression of which of these genes are PARylation-dependent.

## Methods

### Animals

We used 8–9 weeks old C57BL/6 J mice (Envigo, Israel). We included equal numbers of both male and female mice for all the experiments. Mice had ad libitum access to standard chow and drinking water and were kept at constant light–dark cycles for 12 h each, at 22℃.

All animal experiments were approved by the Institutional Animal Care and Use Committee of Ariel University, Israel (approval No. AU-IL-225–07–21 and AU-IL-2407–110). Institutional guidelines were followed for the proper and humane use of animals in research.

### Force Swim Test (FST)

The forced-swim test (FST) was used to induce acute stress. Mice were gently put in a glass cylinder (height: 30 cm, diameter: 10 cm) filled with water (22–33℃) with no option to escape for 6 min. Next, the mice were removed from the cylinder and dried in a warm, dry cage for several moments. The mice were euthanized by cervical dislocation at the following time points after FST: 15 min, 60 min, and 24 h. Naïve mice not introduced to the FST were used as a control group. Cortical tissues were immediately removed, snapped frozen in liquid nitrogen, and then stored at −80℃ for later use.

### Systemic ABT-888 administration

ABT-888 (Veliparib®) is a selective PARP1 and PARP2 inhibitor with higher efficacy towards PARP1 (Thorsell et al. 2017), which crosses the brain-blood-barrier (Donawho et al. 2007). We dissolved ABT-888 hydrochloride in saline and injected 15 mg/kg of ABT888 intraperitoneally at 0.1 ml volume. The injection took place 50 min before the FST based on pharmacokinetic experiments (Donawho et al. 2007) and as we reported before (Lax et al. 2017). Equal volumes of saline were injected to control mice. For a subset of mice, twenty-four hours after the first FST, a second FST was done to assess changes in the duration of immobility as a measure of the adaptive stress response as described before (Gutièrrez-Mecinas et al. 2011; Reul 2014; Saunderson et al. 2016; West 1990). The experiment was video-recorded, and immobility time was manually quantified by a trained experimenter blind to the experimental conditions.

### Protein extraction and western blot

Protein extraction and immunoblotting protocols were done as we described before (Lax et al. 2018). Briefly, frozen cortex samples were lysed using RIPA buffer, loaded into 7% SDS-PAGE gel, and transferred to a nitrocellulose membrane. Membranes were first blocked with 5% non-fat milk in TBST for 1 h and then probed with antibodies against Parp1 and PAR. Beta-actin was used as a reference protein for loading control normalization. On completing the FST, brains were immediately removed to measure Parp1 levels and Parp1 activation (i.e., PARylation) by western blot. Detailed descriptions of the primary and secondary antibodies are provided in Supplemental Table 1. Anti-Parp1, anti-PAR, and anti-beta-actin primary antibodies were diluted 1:1000. The secondary goat anti-mouse antibody was diluted 1: 5000. To detect protein levels, membranes were exposed to an ECL solution, and images were collected using a GelDoC device (Bio-Rad). Image quantification was done with ImageJ software.

**Table 1:** Antibodies that were used for Western Blot. Anti-PAR antibody was used also for ChIP. Anti acetylated histone H4 was used for ChIP

**Antibody Type**	**Target**	**Antibody**	
**Primary**	PAR	Enzo, No.07062114	Monoclonal mouse anti-PAR, clone 10H, Enzo Life-Science, ALX-804–220-R100
	PARP-1	Bio-Rad No.1806	Monoclonal mouse anti-Poly(ADP-Ribose) Polymerase-1 antibody, clone A6.4.12, Bio-Rad, MCA1522G
	Beta-actin	Merck, No.3419587	Monoclonal mouse anti-beta-antibody, clone 4C2, Sigma-Aldrich, MABT825
	Acetylated histone H4	Sigma-Aldrich, No.06–866	Polycolonal rabbit anti-acetyl-Histone H4
**Secondary**	Goat anti mouse IgG/IgM HRP	Merck No.3536068	Goat anti-mouse IgG/IgM HRP-conjugated, EMD Millipore, AP130P

### RNA extraction, reverse transcription, and quantitative PCR (q-PCR)

We extracted cortical RNA using an RNeasy mini kit (Qiagen) following the manufacturer’s protocol. Next, we used GoScript Reverse Transcription Mix, Oligo (dT) kit (Promega, #A2791) to reverse transcribe the RNA into cDNA following the manufacturer protocol.

The qPCR procedure was done using the Hy-syber power mix (Hylabs, Israel) and included primers for the genes listed in supplemental Table 2. RNA expression levels were normalized to beta-actin expression levels, and values were calculated using the ∆∆CT method.

**Table 2:** Primer sequences used to perform q-PCR and q-ChIP

Egr1	Forward	CAATCCTCAAGGGGAGCCG
Egr1	Reverse	GAGCGATGTCAGAAAAGGACTCTGT
Egr2	Forward	TCACAGGCAGGAGAGAGTCA
Egr2	Reverse	GCTGTCAGGCAGCTGGAG
Egr3	Forward	GACAATCTGTACCCCGAGGAG
Egr3	Reverse	GTCCATCACATTCTCTCTCCC
Nr4a1	Forward	GCGAAAGTTGGGGGAGTGTG
Nr4a1	Reverse	TGAATACAGGGCATCTCCAGCC
C-Fos	Forward	TACTACCATTCCCCAGCCGA
C-Fos	Reverse	CTGCGCAAAAGTCCTGTGTG
Arc	Forward	CACTCTCCCGTGAAGCCATT
Arc	Reverse	GGTGCCCACCACATACTGAA
Nrn1	Forward	CAGCACACTGTTTCTCAGACC
Nrn1	Reverse	AAAACCCTGTCACGTCTCGC
Bdnf	Forward	TTGTTTTGTGCCGTTTACCA
Bdnf	Reverse	GGTAAGAGAGCCAGCCACTG
β-actin	Forward	GGCTGTATTCCCCTCCATCG
β-actin	Reverse	CCAGTTGGTAACAATGCCATGT
Arc promoter (ChIP)	Forward	GAGGAGCTTAGCGAGTGTGG
Arc promoter (ChIP)	Reverse	GCAGCATAAATAGCCGCTGG
Egr1 promoter (ChIP)	Forward	CTCCCAGTTGGGAACCAAGG
Egr1 promoter (ChIP)	Reverse	TATATGGCGTTTCCGGGTCG
Egr2 promoter (ChIP)	Forward	5'-CGGGACAAAGCTCACACTCT-3'
Egr2 promoter (ChIP)	Reverse	5'-GCAACGGTCTTGTGAGCATC-3'
Egr3 promoter (ChIP)	Forward	5'-GCTTTGCATTCGGCTTCGTC-3'
Egr3 promoter (ChIP)	Reverse	5'-TTAGAAAGCAGGAAGCGGGC-3'
C-Fos promoter (ChIP)	Forward	5'-GCCCAGTGACGTAGGAAGTC-3'
C-Fos promoter (ChIP)	Reverse	5'-GTCGCGGTTGGAGTAGTAGG-3'

## Quantitative chromatin immunoprecipitation (Q-ChIP)

Mice were sacrificed, and cortices were rapidly isolated, flash-frozen, and stored at-80 °C for later analysis. Samples were homogenized in 1 X PBS including 1% formaldehyde, and the homogenates were kept for 10 min at room temperature with gentle agitation. Cross-linking reactions were stopped by adding glycine (125 mM) for 10 min at room temperature with gentle agitation. Fixed chromatin samples were then homogenized in cell lysis solution (PIPES 5 mM (pH 8), KCl 85 mM, NP40 0.5%) and centrifuged for 5 min at 3000 rpm, 4 °C. Pellets were resuspended in RIPA-light solution (NaCl 150 mM, SDS 0.3%, Tris–HCl 50 mM (pH 8)) and sonicated to a size range of ~ 200–500 bp fragments using a sonics-vibracell-vcx750 sonicator with the following parameters: 30% power, in cycles of 10 s on/10 s off, for a total of 36 min on ice. Sonicated chromatin samples were centrifuged for 2 min at full speed at 4 °C. Pellets were resuspended in 1 ml of RIPA-light solution. Chromatin samples were pre-cleared with 10 µl of Magna-ChIP® Protein-G magnetic beads (EMD Millipore, Cat: 16–662) pre-blocked with BSA. Next, 20 µg of chromatin were incubated overnight at 4 °C with an anti-PAR antibody or anti-acetylated H4 antibody (2 µg and 4 µg respectively, see Supplementary Table 1). Input controls were treated the same way except for not adding an antibody to the solution. Antibodies and chromatin were mixed with 20 µl of Magna-ChIP® Protein-G magnetic beads (EMD Millipore, Cat: 16–662) for 2 h at 4 °C. The beads were then washed with low-salt wash buffer (5 min; 0.1% SDS, 1% Triton-X, 2 mM EDTA, 20 mM Tris 150 mM NaCl), then with high-salt wash buffer (5 min, 0.1% SDS, 1% Triton-X, 2 mM EDTA, 20 mM Tris 500 mM NaCl) and then with wash-solution (5 min; 0.25 M LiCl 1% NP-40 1% deoxycholate 1 mM EDTA 10 mM Tris, pH 8.0) followed by six washes with TE (5 min each). Protein–DNA complexes were eluted from the beads with elution buffer (1% SDS 0.1 M NaHCO3), de-cross-linked, treated with proteinase K, and purified. Purified DNA was resuspended in 100ul elution buffer. For QPCR analysis, SYBR green quantitative PCR was performed (primer sequences are listed in supplementary Table 2). To determine the relative enrichment of PAR and acetylated H4, the 2 -ΔΔCt method was used with normalization to the input data.

## Statistics

Data are expressed as mean ± SEM unless otherwise stated. The data were analyzed by one-way ANOVA followed by Tukey’s post-hoc tests. For ABT888's effect on immobility in the FST tests, two-way repeated measures ANOVA with time and treatment were the main factors followed by pair-wise Bonferroni-corrected t-tests. Comparisons between two groups in the qPCR and Q-ChIP experiments were made with the ∆∆CT method and one-sample two-tailed t-tests. P-values smaller than 0.05 were considered statistically significant. Possible outliers were visible in the data; therefore, we applied the Iglewicz and Hoaglin's test for outliers under the strict criterion of z-score ≥|3.5|. Data analysis of the immobility time in the FST found no effect on sex; therefore, we combined the data from both sexes for all analyses.

## Results

### FST triggers elevated cortical PARylation

**Fig. 1:**
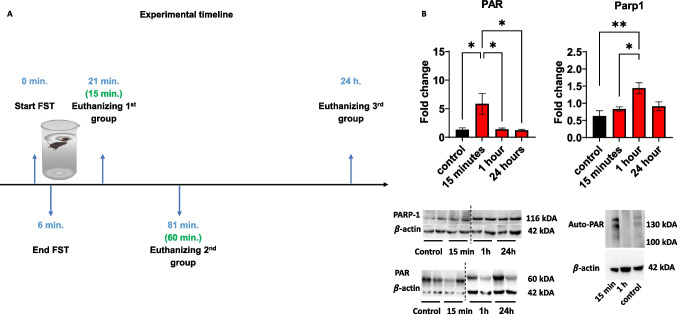
**A** Experimental timeline: Force Swimming Test and euthanization of the experimental groups; **B** Expression levels of cortical PAR and PARP1 at the time points described in (A). One-way ANOVAs for PAR levels: F (3,18) = 5.243, p = 0.0089, followed by Tukey’s post-hoc test (control *n* = 5, 15 min *n* = 6, 1 h n = 6, 24 h *n* = 6); one-way ANOVA for Parp1 levels: F (3,20) = 6.748, *p* = 0.0025, followed by Tukey’s post-hoc test. *n* = 6 for all groups. *p < 0.05, **p < 0.01

Mice were exposed to the FST for 6 min and were sacrificed 15 min, 1 h, or 24 h later (Fig. 1A). Cortical PARylation, including auto-PARylation of Parp1, was rapidly elevated 15 min after the FST and then returned to basal levels at 1 h post-FST and remained at those level 24 h later (Fig. 1B; left). This elevated PARylation was followed by a slower increase in Parp1 levels, the primary enzyme that catalyzes PAR, 1-h post-FST. Parp1 levels were reduced 24 h later (Fig. 1B; right). These findings suggest that FST triggers PARylation, in line with previous studies that showed neuronal- and neurotrophic-activity-induced PARylation (Azad et al. 2018; Homburg et al. 2000; Visochek et al. 2005).

### FST induces upregulation of cortical immediate-early genes

**Fig. 2:**
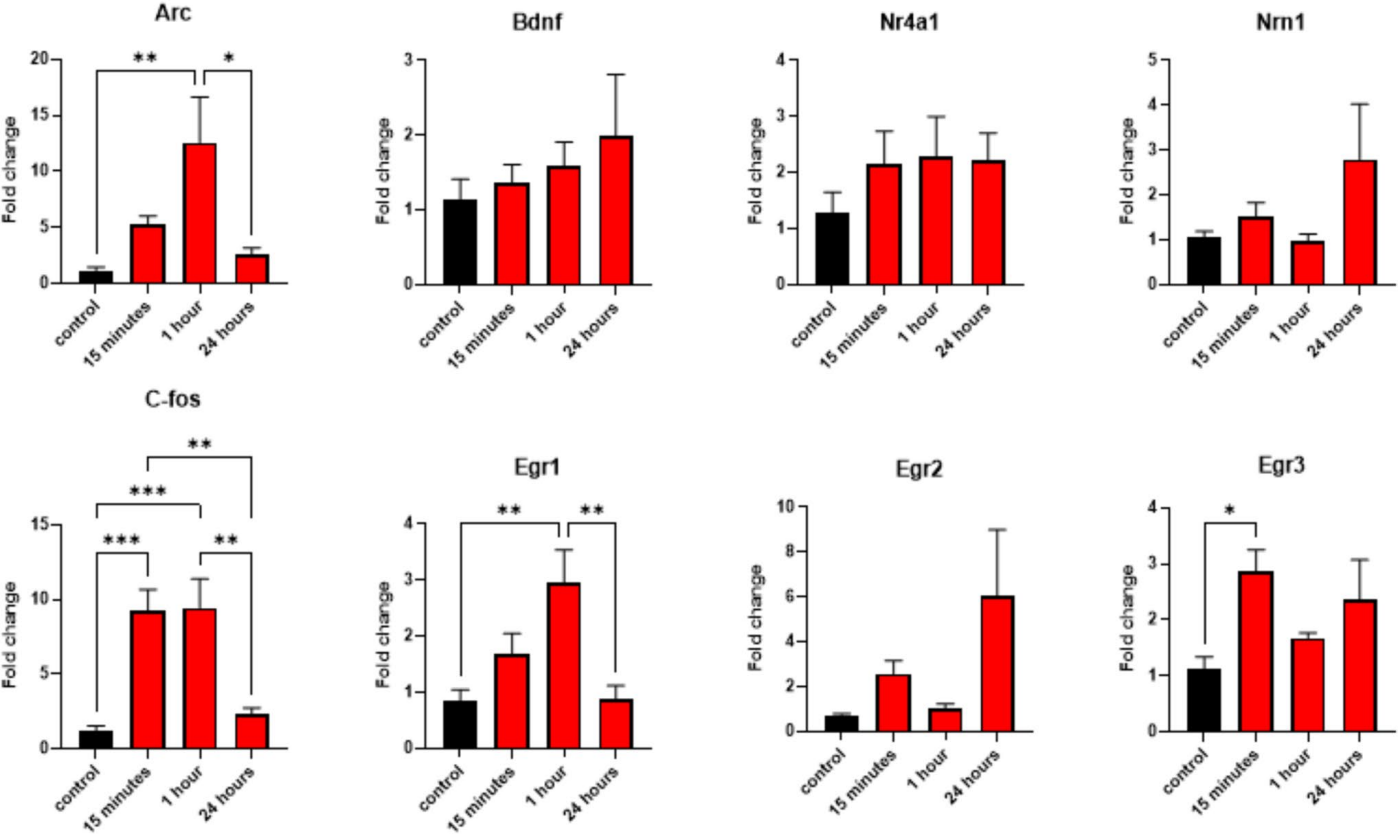
Expression levels of immediate-early and activity-dependent genes at several time points following FST. One-way ANOVAs for Arc: F (3, 20) = 5.991, *p* = 0.0044; Bdnf: F (3, 20) = 0.5675, *p* = 0.6428; Nr4a1: F (3, 20) = 0.7207, *p* = 0.5513; Nrn1: F (3, 20) = 1.741, *p* = 0.1909; C-Fos: F (3, 20) = 12.96, *p* < 0.0001; Egr1: F (3, 20) = 7.051, *p* = 0.0020; Egr2: F (3, 20) = 2.578, *p* = 0.0823; Egr3: F (3, 20) = 3.400, *p* = 0.0378. Followed by Tukey’s post-hoc tests. n = 6 for all groups. *p < 0.05, **p < 0.01, ***p < 0.001

Since FST causes extensive neuronal activation in the cortex (Connor et al. 1997; Slattery et al. 2011), we examined cortical mRNA expression levels of several immediate-early genes known to be transcribed upon neuronal activation. The mRNA expression levels were measured at the same time points as the PARylation levels (15 min, 1 h, or 24 h post-FST). We found a significant increase in Arc, c-Fos, Egr1, and Egr3 mRNA levels 15 min to 1 h after the FST. The mRNA levels returned to baseline levels 24 h later, and in the case of Egr3, mRNA levels returned to baseline levels already after 1 h (Fig. 2). Egr2, Bdnf, Nrn1, and Nr4a1 did not show a significant increase in mRNA levels, although, in all these genes, a trend toward increased mRNA expression was observed (Fig. 2).

### Parp inhibition impairs stress-coping behavior in the FST

**Fig. 3:**
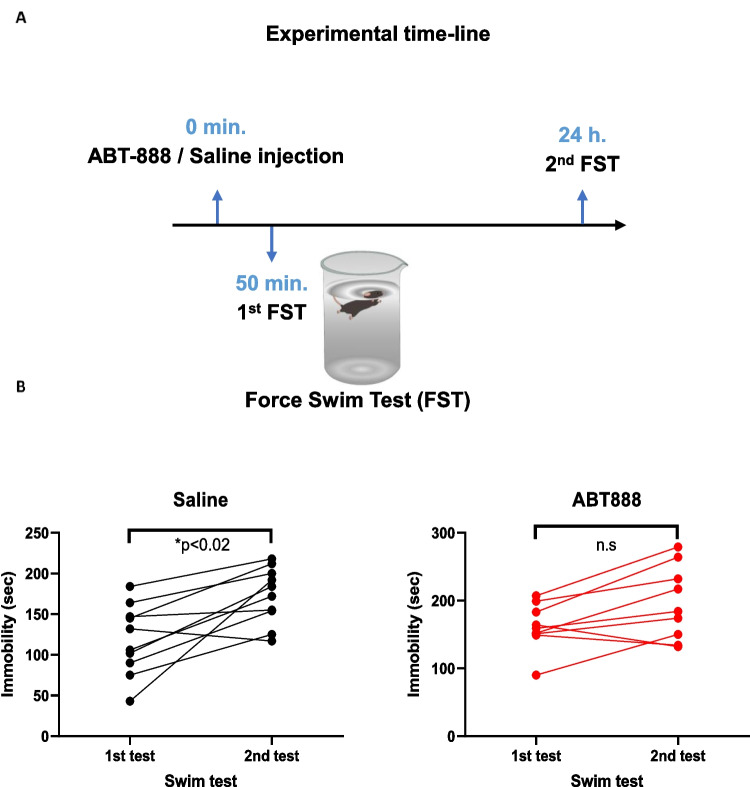
**A** Experimental timeline: ABT-888 / saline injection, followed by two FSTs, 24 h apart. **B** Immobility time (in seconds) for each of the FSTs. Two-way repeated-measures ANOVA with a main effect of time F(1,17) = 21.09, p = 0.0003, no main effect of treatment F(1,17) = 3.81, *p* = 0.0675 and no interaction F(1,17) = 1.01, *p* = 0.3288, followed by pair-wise Bonferroni-corrected t-tests. *n* = 10 for the saline group and *n* = 9 for the ABT888 group. *p < 0.02; n.s: no significant

Next, we explored whether elevated PARylation induces the commonly seen shift in stress coping strategy from swimming as an active strategy into floating (i.e., immobility) as a passive strategy (Commons et al. 2017). For this aim, we injected the mice with the Parp inhibitor ABT888 (15 mg/kg IP, total volume of 0.1 ml) or saline as a vehicle 50 min before the first FST. Twenty-four hours later, we re-exposed the mice to a second FST and recorded the immobility time during these two FST sessions (Fig. 3A). We found that ABT888-injected mice showed no significant increase in immobility time, while control mice did show this shift in behavior (Fig. 3B). This finding suggests that PARylation promotes the observed shift in stress coping strategy, likely through gene-expression regulation of immediate-early genes.

### Parp inhibition reduces Arc expression after FST

**Fig. 4:**
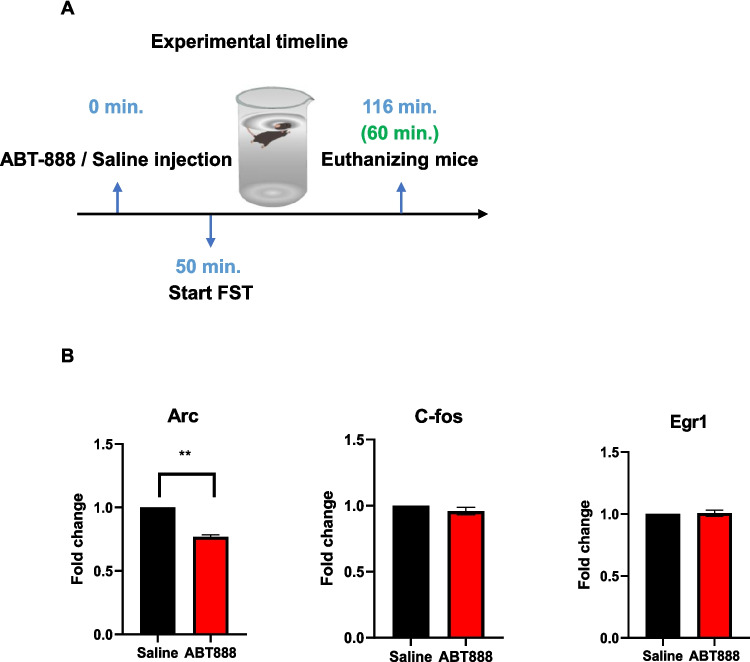
**A** Experimental timeline: ABT-888 / saline injection, followed by FST and mice euthanization 1 h after the end of the FST. **B** The relative expression level of the genes elevated 1 h after FST: Arc, C-fos, and Erg-1. T-test, **p < 0.01, Arc: saline *n* = 11, ABT888 *n* = 14; c-Fos: saline *n* = 12, ABT888 *n* = 15; Egr1: saline *n* = 12 ABT888 = 12

Based on our findings, we speculated that the FST-induced increase in mRNA expression of at least some of the immediate-early genes is due to chromatin PARylation and, hence, relaxation that enables transcription. To identify which of the immediate-early genes were regulated through PARylation, we repeated the injection of ABT888 with the same settings, introduced the mice to the FST, and one hour later, we measured cortical mRNA levels of the immediate-early genes that were significantly elevated in the previous experiment (i.e., Arc, C-Fos, Egr1; Fig. 4A). We found that Arc (but not c-fos and Egr1) mRNA expression was significantly reduced following FST due to Parp inhibition (Fig. 4B). This implies that Arc is a downstream target of FST-induced PARylation.

### Parp inhibition reduces chromatin PARylation at the Arc promoter after FST

**Fig. 5:**
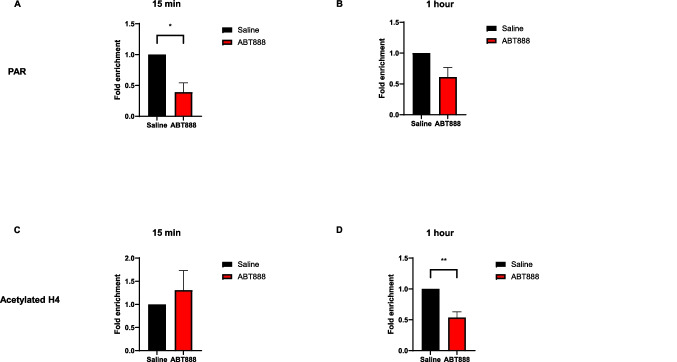
Enrichment of PARylation and acetylated H4 on the Arc promoter. PAR enrichment on the Arc promoter was significantly reduced 15 min **A** but not 1 h **B** after the FST in ABT888 pretreated mice. T-test, *p < 0.05, n = 6 per group for the 15-min experiment and saline n = 4, ABT888 n = 3 for the 1-h experiment. **C-D** A significantly reduced acetylation of histone H4 was observed on the Arc promoter 1 h (**D**) but not after 15 min (**C**) after the FST in ABT888 pretreated mice. T-test, **p < 0.01, saline *n* = 3, ABT888 *n* = 6 for the 15-min experiment and n = 6 per group for the 1-h experiment

Next, to establish that PARylation directly regulates Arc mRNA expression, we injected ABT888 as before and introduced the mice to the FST. We sacrificed the mice 15 min or 1 h later and extracted the cortices. We performed quantitative chromatin immunoprecipitation (qChIP) of PAR followed by qPCR of the Arc promoter region. We found that 15 min after the swim test, mice pretreated with ABT888 showed significantly lower PARylation levels on the Arc promoter compared to saline-pretreated controls (Fig. 5A). This effect was not seen 1 h after the swim test (Fig. 5B), in line with the transient nature of PARylation we observed before. These findings suggest that Arc is a PARP-dependent gene activated during the FST task.

Egr1 and Egr3 (but not Egr2) are transcription factors that directly target the Arc gene 2- to 4 h post-stimulation (Li et al. 2005). Therefore, we performed qChIP on the promoter regions of the Egr1-3 genes as well. We found ABT888 to significantly reduce chromatin PARylation at the Egr2 promoter 15 min, but not 1 h after FST. We did not find any significant effect at the promoters of Egr1 and Egr3 at both time points (supplementary Fig. 1). Since Egr2 does not target the Arc promoter and its expression levels did not change significantly after FST, Egr2 is likely not directly regulating cortical Arc expression shortly after FST.

As previous findings showed (Visochek et al. 2016), Parp inhibition reduces core histone acetylation in the promoter region of several immediate-early genes, we therefore performed qChIP on the promoter regions of Arc, cFos, and Egr1 using an antibody against the acetylated core histone H4. We found that Parp inhibition reduced acetylation of H4 near these promoters 1 h, but not 15 min, after FST (Fig. 5C-D, supplementary Fig. 2). These findings align with the idea that histone PARylation is a rapid event that precedes and enables the acetylation of core histones to maintain increased expression of immediate-early genes.

## Discussion

Neuronal and behavioral responses to stressful events are required for proper adaptation to stress. However, the underlying molecular mechanisms that govern this response are yet to be fully explored. Here, we showed that PARylation, a necessity for long-term memory formation (Cohen-Armon et al. 2004), is induced shortly after exposure to FST and promotes adaptive stress coping.

PARylation happens shortly after neuronal activation or a neurotrophic stimulus (Homburg et al. 2000; Visochek et al. 2005; Wang et al. 2012). Parp1 catalyzes PAR chain formation on itself (i.e., auto-PARylation) and nearby histones, thus likely increasing chromatin accessibility (Azad et al. 2018; Kraus & Lis 2003). This, in turn, enables elevated gene expression of immediate-early genes. An earlier *in-vitro* study found that high-frequency stimulation of cultured rat cerebral neurons induced PARylation-mediated expression of Arc, c-Fos, and Egr1 ( Visochek et al. 2016). However, whether PARylation-mediated expression of these and other immediate-early genes occurs *in-vivo* in response to stressful stimuli was unknown. We hypothesized that (a) PARylation would be induced upon FST as part of the expected cortical neuronal response and stress adaptation; (b) that transcription of neuronal activity-dependent immediate-early genes that promotes neuronal plasticity and long-term memory formation are likely targets of this FST-induced PARylation (Cohen-Armon et al. 2004; Goldberg et al. 2009; Hernández et al. 2009; Visochek et al. 2016); and (c) that Parp inhibition will reduce the expression of PARP-dependent genes upon FST, resulting in reduced immobility in the second FST due to either impaired adaptation response to the stress induced by the first FST or impaired memory formation of the first FST.

As expected, we found that FST rapidly induced elevated cortical PARylation 15 min after FST, accompanied by a slower increase in Parp1, the primary enzyme that catalyzes PARylation. Further, systemic Parp1 inhibition diminished adaptive stress response in a second FST test. Gene-expression analysis of genes that were elevated after FST revealed that Parp1 inhibition reduced the expression of Arc, a known regulator of synaptic plasticity and protein-synthesis-dependent long-term potentiation (Korb & Finkbeiner 2011; Messaoudi et al. 2007). Also, using chromatin immunoprecipitation, we demonstrated ABT888 successfully reduced chromatin PARylation at the Arc gene promoter after FST followed by reduced acetylation of H4, suggesting PARylation promotes histone acetylation and has a regulatory role on Arc expression. These findings partly recapitulate previous *ex-vivo* work demonstrating a PARylation-dependent increase in Arc, C-Fos, and Egr1 expression following high-frequency electrical stimulation of rat cortical neurons (Visochek et al. 2016). We did not detect a PARylation-dependent increase in C-fos or Egr1 gene-expression, although we did observe reduced histone 4 acetylation near their promoters 1 h after FST in ABT888 pretreated mice. This partial discrepancy between the two studies may be due to the different experimental settings and species used (embryonic neuronal cell culture in rats vs. cortical tissue in adult mice). It is also possible that another gene regulatory mechanism was over-activated at the promoters of cFos and Egr1 in ABT888 pretreated mice as a compensatory mechanism for stress adaptation. Future unbiased proteomic and biochemical experiments will be required to assess which proteins are PARylated and which other post-translational histone modifications are induced due to PARylation. Some candidates include PARylation of histone 1 (Goldberg et al. 2009; Visochek et al. 2016) and eviction of histone 1 (Azad et al. 2018). Such studies will improve our understanding of the molecular events downstream of PARP1 activity and chromatin PARylation following FST.

In addition, while Arc was found to be upregulated in a PARP-dependent manner in our model and is essential for memory consolidation (Plath et al. 2006), we note that to unequivocally prove Arc is necessary for adaptive stress-coping behavior, acute Arc inhibition rapidly after the first FST will be required. This is currently hard to achieve as there are no Arc-specific inhibitors available.

Intra-cerebral and systemic PARP inhibitor applications were done before to manipulate reconsolidation and extinction of fear conditioning successfully (Elharrar et al., 2021; Goldberg et al. 2009; Inaba et al. 2015). However, the downstream effects of this inhibition were unknown. Our findings expand the role of PARylation in modulating behaviors to include stress coping. Further, we filled the gap in knowledge between the behavioral effects of PARP inhibition and the observed PARylation-dependent long-term potentiation and increased expression of some immediate-early genes after *ex-vivo* stimulation. It is important to consider, however, that the FST is a stressful learning experience. Previous studies have shown that some immediate-early genes are activated by stress alone, learning alone, or both (Allen et al. 2018). Since the FST is not designed to separate which immediate-early genes were upregulated by the stress or learning components of the task, the changes in gene expression should not be interpreted as strictly stress- and/or learning-induced but rather as FST-induced.

Our experiments were focused on several potential target genes; further research is needed to explore the transcriptome-wide effects of FST-induced PARylation and PARP inhibition using RNA-sequencing or other high-throughput approaches. In addition, we examined the impact of FST and PARP inhibition in the cerebral cortex to closely follow the tissue selection done in previous reports (Fontán-Lozano et al. 2010; Goldberg et al. 2009; Visochek et al. 2005). Future studies are required to decipher the effects of FST on PARylation and gene expression in different subregions of the cortex and other relevant brain regions, such as the hippocampus and the amygdala. We recognize these limitations and present our findings as a first step towards understanding the PARylation-dependent mechanisms of activity-dependent gene expression regulation required for proper stress-coping behavior.

## Supplementary Information

Below is the link to the electronic supplementary material.

Supplementary file1 (PPTX 99 KB)

Supplementary file2 (PPTX 138 KB)
