# Supplementary material for: Poly ADP‐ribosylation regulates Arc expression and promotes adaptive stress-coping
Source: Psychopharmacology (Berl). 2025 Jan 14;242(4):741–50. doi: 10.1007/s00213-025-06744-8 (PMC11890342; doi:10.1007/s00213-025-06744-8)
Supplement: Supplementary file 1 — Supplementary file1 (PPTX 99 KB) [file 213_2025_6744_MOESM1_ESM.pptx]

## Slide 1
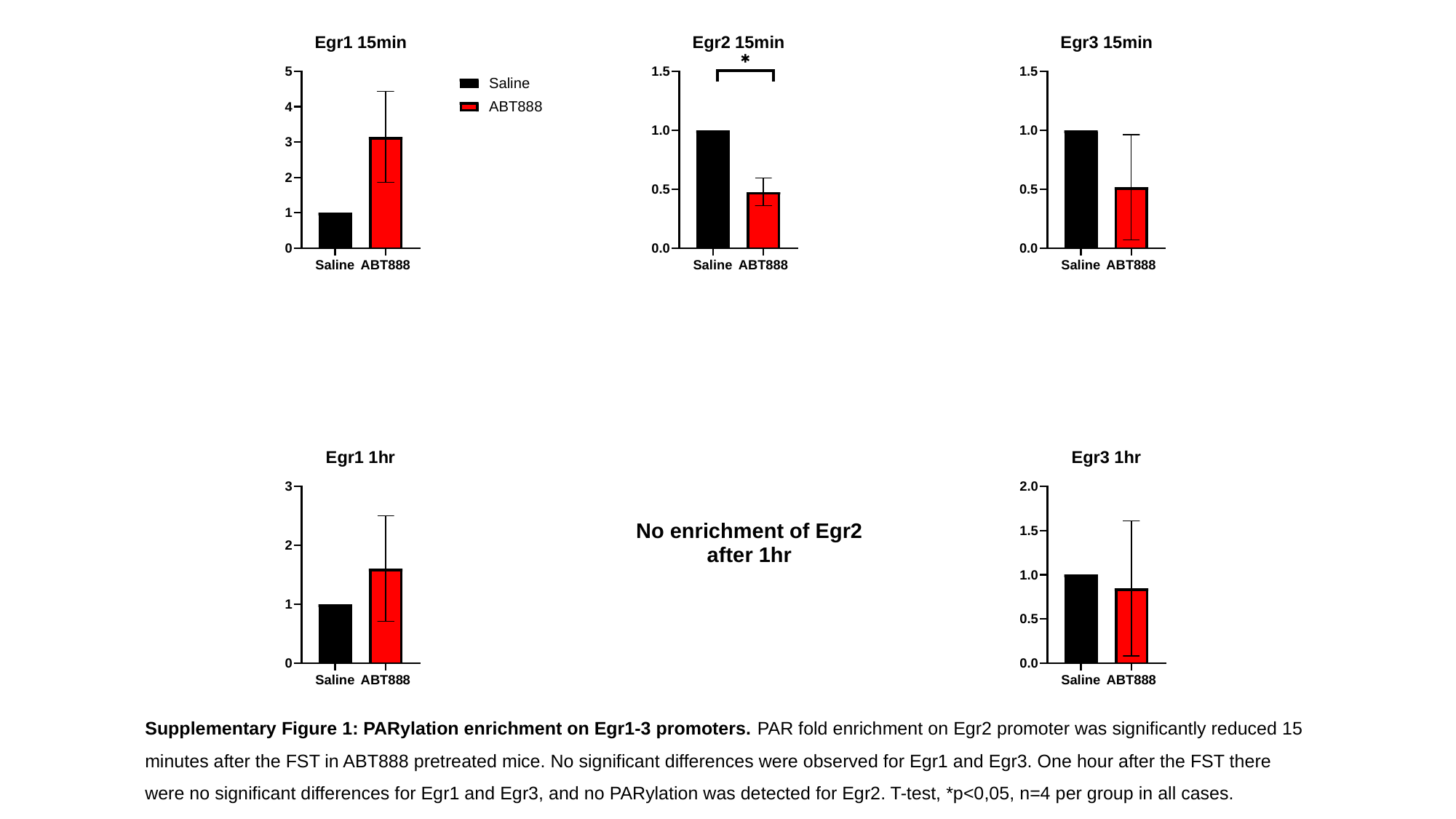

Supplementary Figure 1: PARylation enrichment on Egr1-3 promoters. PAR fold enrichment on Egr2 promoter was significantly reduced 15 minutes after the FST in ABT888 pretreated mice. No significant differences were observed for Egr1 and Egr3. One hour after the FST there were no significant differences for Egr1 and Egr3, and no PARylation was detected for Egr2. T-test, *p<0,05, n=4 per group in all cases.
