# Supplementary material for: Poly ADP‐ribosylation regulates Arc expression and promotes adaptive stress-coping
Source: Psychopharmacology (Berl). 2025 Jan 14;242(4):741–50. doi: 10.1007/s00213-025-06744-8 (PMC11890342; doi:10.1007/s00213-025-06744-8)
Supplement: Supplementary file 2 — Supplementary file2 (PPTX 138 KB) [file 213_2025_6744_MOESM2_ESM.pptx]

## Slide 1
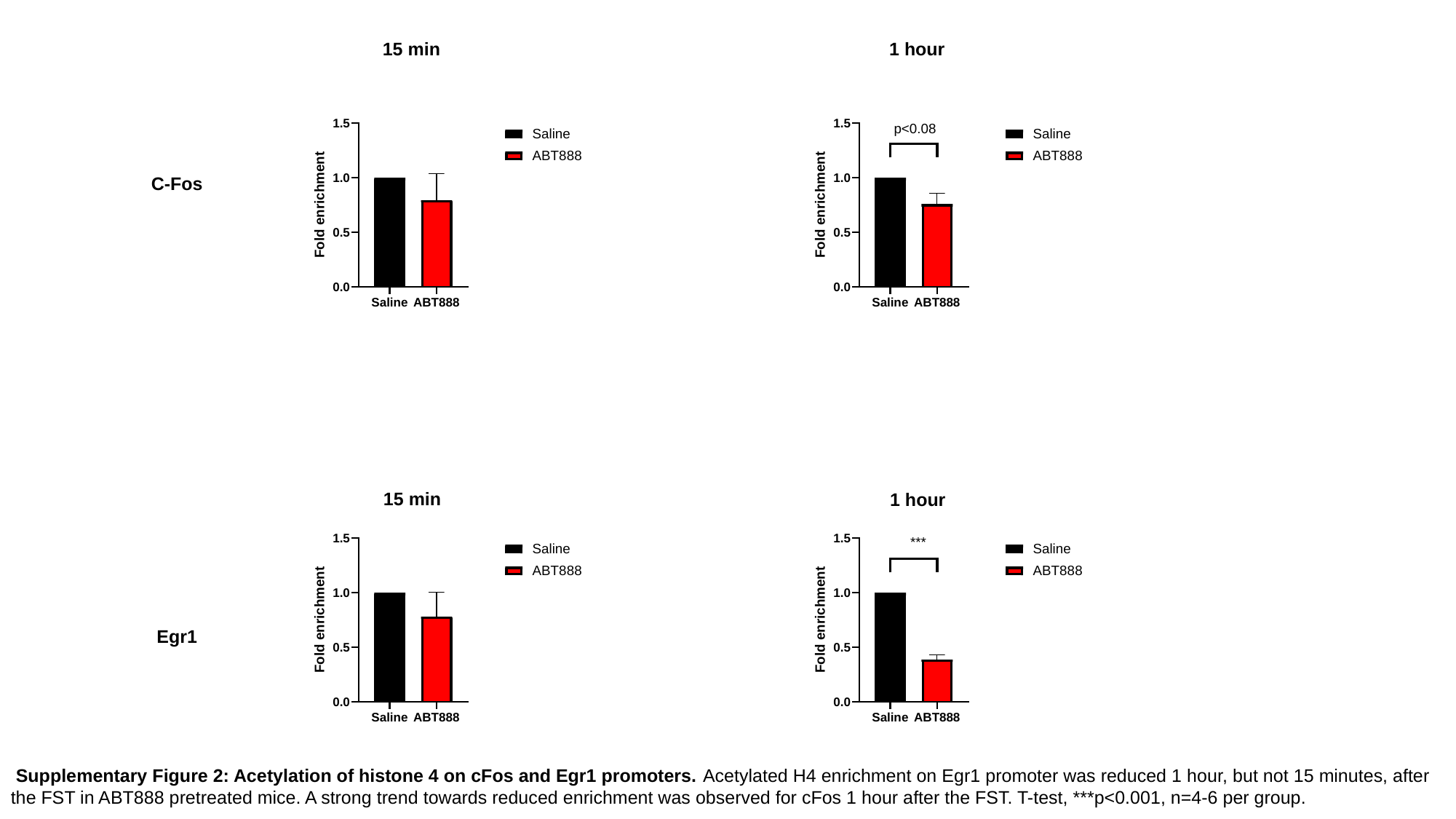

15 min
1 hour
C-Fos
15 min
1 hour
Egr1
 Supplementary Figure 2: Acetylation of histone 4 on cFos and Egr1 promoters. Acetylated H4 enrichment on Egr1 promoter was reduced 1 hour, but not 15 minutes, after the FST in ABT888 pretreated mice. A strong trend towards reduced enrichment was observed for cFos 1 hour after the FST. T-test, ***p<0.001, n=4-6 per group.
